# Clinical utility of the C‐reactive protein:albumin ratio in non‐small cell lung cancer patients treated with nivolumab

**DOI:** 10.1111/1759-7714.13788

**Published:** 2021-01-12

**Authors:** Taisuke Araki, Kazunari Tateishi, Kei Sonehara, Shuko Hirota, Masamichi Komatsu, Manabu Yamamoto, Shintaro Kanda, Hiroshi Kuraishi, Masayuki Hanaoka, Tomonobu Koizumi

**Affiliations:** ^1^ First Department of Internal Medicine Shinshu University School of Medicine Matsumoto Japan; ^2^ Japan Red Cross Society Nagano Red Cross Hospital Nagano Japan; ^3^ Department of Comprehensive Cancer Therapy Shinshu University School of Medicine Matsumoto Japan

**Keywords:** C‐reactive protein:albumin ratio, immunotherapy, inflammatory marker, non‐small cell lung cancer

## Abstract

**Background:**

Nivolumab is a second‐line chemotherapy for non‐small cell lung cancer (NSCLC). This study explored the impact of clinical biomarkers such as neutrophil:lymphocyte ratio (NLR), C‐reactive protein:albumin ratio (CAR), and modified Glasgow prognostic score on the efficacy and outcome of nivolumab monotherapy in previously treated NSCLC patients.

**Methods:**

We retrospectively analyzed advanced or postoperative recurrence of NSCLC in 113 patients in two Japanese facilities from January 2015 to December 2019. Optimal cutoff values of NLR and CAR were assessed by the area under the receiver operating characteristic curves predicting death events to conduct regression analysis. Baseline values and values collected eight weeks after nivolumab treatment were measured to investigate time‐series changes of these markers.

**Results:**

The patients showed median overall survival (OS) and progression‐free survival (PFS) of 14.0 months and 2.3 months, respectively, with both being significantly longer in patients with partial response (PR) than in patients with progressive disease (PD). Optimal cutoff levels for NLR and CAR were 5.8 and 0.83, with significant decrease in CAR (*P* = 0.002) from baseline levels in PR patients and significant increase in PD patients. Baseline CAR ≥0.83 was significantly associated with one‐year mortality events and overall survival (OS), and multivariate analysis showed significant association of age ≤70 years, an Eastern Cooperative Oncology Group performance status score of 2 or 3, and a baseline CAR ≥0.83 with inferior OS.

**Conclusions:**

For second‐line nivolumab therapy, evaluation of baseline CAR and subsequent changes in CAR may be predictive of therapeutic response to nivolumab and long‐term survival in NSCLC patients.

**Key points:**

**Significant findings of the study**

The baseline value of C‐reactive protein:albumin ratio was significantly associated with one‐year mortality and overall survival in non‐small cell lung cancer patients treated with nivolumab.

**What this study adds**

Time‐series change of C‐reactive protein:albumin ratio may be useful for predicting the treatment efficacy in patients treated with nivolumab.

## Graphical abstract

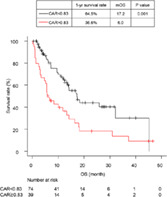

The baseline C‐reactive protein:albumin ratio was significantly associated with overall survival in non‐small cell lung cancer patients treated with nivolumab monotherapy. The time‐series change of this marker may be useful in predicting the treatment efficacy with nivolumab.

## Introduction

Immune checkpoint inhibitors (ICIs) are new types of anticancer agents with a mechanism different from that of conventional agents and have significantly improved clinical outcomes for non‐small cell lung cancer (NSCLC) patients. Nivolumab is a programmed death‐1 (PD‐1) antibody, approved as a standard second‐line chemotherapy for NSCLC in 2015 following two randomized, open‐label, phase III clinical trial in patients with advanced squamous^1^ or nonsquamous^2^ NSCLC, in which prolonged overall survival (OS) and a favorable safety profile relative to docetaxel therapy has been demonstrated. The five‐year OS rate for nivolumab is 16%,^3^ superior to the historic five‐year OS rates for advanced NSCLC (1%–8%).^4^, ^5^ These results identified long‐term responders to nivolumab therapy as compared to responders with a 20% response rate to nivolumab,^1^, ^2^, ^6^ with approximately one half of patients showing disease progression in response to initial treatment.^1^, ^2^ However, the clinical backgrounds and laboratory characteristics of long‐term responders or survivors following nivolumab therapy have not been well described.

Inflammation status plays an important role in carcinogenesis. Specifically, the tumor microenvironment regulated by various inflammatory cells plays an essential role in the neoplastic process.^7^ White blood cells or their fractions are typical hematological parameters that reflect inflammatory conditions. Additionally, the neutrophil:lymphocyte ratio (NLR), lymphocyte:monocyte ratio, and platelet:lymphocyte ratio calculated according to the ratio of complete blood count (CBC) have been investigated for their prognostic value in various cancers.^8^, ^9^, ^10^ Moreover, serum C‐reactive protein (CRP) and albumin are important inflammatory markers, and the C‐reactive protein:albumin ratio (CAR) is a prognostic factor in many cancers.^11^ The modified Glasgow prognostic score (GPS) comprising serum CRP, albumin, as well as CAR measurements, indicates prognostic value in several cancers, including lung cancer.^12^ These parameters reflect the dynamic balance between antitumor immunity and the tumor‐promoting environment affected by the inflammatory response. These prognostic inflammation‐based parameters have been investigated for their predictive or prognostic utility in numerous studies with regard to immunotherapy application in NSCLC;^13^, ^14^ however, the findings are controversial, and a conclusive efficacious parameter has not yet been identified.

In the present study, we investigated clinical parameters and biomarkers to identify their clinical utility by evaluating their predictive or prognostic efficacy in NSCLC patients treated with nivolumab monotherapy.

## Methods

### Study population and data collection

This retrospective study was approved by the Institutional Review Board of Shinshu University School of Medicine (approval no. 4656, Matsumoto City, Japan) and conducted in accordance with the principles of the Declaration of Helsinki. We conducted a retrospective analysis of 116 patients with a pathological diagnosis of NSCLC, who were treated with nivolumab as second‐line therapy at two institutes (Shinshu University Hospital: an academic medical center in Matsumoto, Japan, and Nagano Red Cross Hospital: a community hospital in Nagano, Japan) from January 2015 to December 2019. All patients met the following criteria: histological or cytological diagnosis of NSCLC, unresectable or advanced‐stage NSCLC (stage III or IVA and IVB) or postoperative recurrence, treatment with at least one cycle of nivolumab monotherapy at a standard dose (3 mg/kg or 240 mg/bodyweight every two weeks), and at least one regimen of chemotherapy with cytotoxic anticancer agents or molecular‐targeting agents (epidermal growth factor receptor (*EGFR*) tyrosine kinase inhibitors or anaplastic lymphoma kinase (*ALK*) inhibitors) prior to nivolumab therapy. Patient data and the results of their clinical course were collected from electronic medical records. Baseline demographic data included age and sex, and clinicopathological data included histology, clinical or pathological stage (according to the eighth edition of the TNM classification for lung cancer), presence of *EGFR* mutations and/or the *ALK* fusion gene, programmed death‐ligand 1 (PD‐L1) tumor proportion score, the number of previous regimens, and performance status (PS) as evaluated by the Eastern Cooperative Oncology Group (ECOG). The baseline laboratory data included peripheral CBC including absolute neutrophil count (ANC) and absolute lymphocyte count (ALC), and serum creatinine, albumin and CRP levels. NLR was calculated by dividing ANC by ALC, and the CAR was calculated by dividing CRP levels by albumin levels. The modified GPS was evaluated according to serum CRP and albumin levels, with scores ranging from 0 to 2 and calculated as follows: CRP ≤1.0 mg/dL = 0, CRP >1.0 mg/dL = 1, CRP >1.0 mg/dL and albumin <3.5 g/dL = 2. Baseline data were defined as the most recent laboratory data collected within one‐week prior to the start of nivolumab therapy. The same data were collected eight weeks after starting nivolumab therapy to investigate time‐series changes. The efficacy of nivolumab was evaluated according to the revised Response Evaluation Criteria in Solid Tumors guidelines (v.1.1),^15^ which allowed calculation of the overall response rate (ORR) and disease control rate (DCR), although clinical evaluation by the original physicians were also adopted. The primary outcome of this study was OS defined as the period from the start of nivolumab therapy to either a fatal event or censored observation. The secondary outcome was one‐year mortality and progression‐free survival (PFS) defined as the period from the start of nivolumab treatment to death or progression.

### Statistical analysis

A receiver operating characteristic (ROC) curve was constructed using pretreatment NLR and CAR as the test variables and with the death events (death or survival and censored events) as the state variables. The optimal cutoff values for NLR and CAR were assessed by calculating the area under the ROC curves (AUCs) for predicting death events in order to perform regression analysis. Kaplan–Meier analysis was performed to plot the OS and PFS curves, and the log‐rank test was employed for intergroup comparison of OS and PFS. Logistic regression analysis was performed to identify the significant variables for one‐year mortality events. A Cox proportional hazards model was used to identify the prognostic factors for OS, with statistically significant variables used for the univariate model, with the clinically important variables further analyzed by multivariate analysis. Time‐series analysis of NLR and CAR was conducted using the Wilcoxon signed‐rank test. All statistical analyses were performed with EZR (Saitama Medical Center, Jichi Medical University, Saitama, Japan), a graphical user interface for R (The R Foundation for Statistical Computing, Vienna, Austria),^16^ and statistical significance was determined as *P* < 0.05.

## Results

### Baseline characteristics

The cohort comprised 116 patients treated with nivolumab in two hospitals, with three patients excluded due to insufficient baseline laboratory tests, resulting in 113 eligible patients. The baseline demographic and clinicopathological characteristics of the patients are shown in Table 1. Their median age was 68.5 years (range: 36–86 years), with 87 (77.0%) males and 26 (23.0%) females. Adenocarcinoma and squamous cell carcinoma accounted for >90% of histological diagnoses, with >80% patients in an advanced stage and 16.3% experiencing postoperative recurrence. The *EGFR* mutation or the *ALK* fusion gene was carried by 18 patients. The PD‐L1 was evaluated in 60 patients (53.1%); and 33 (29.1%) were found to have PD‐L1 positive expression. Also, only five (4.4%) patients had been administered antibiotic therapy at the start of nivolumab. The percentage of poor PS (score 2 or 3) was 18.1%, and nivolumab was a second‐line therapy in approximately 50% patients, whereas it was the fourth‐line therapy that 32.7% patients had received. Additionally, 77 patients had received platinum‐based chemotherapy and 14 patients had received chemoradiotherapy as first‐line therapy. Among the enrolled patients, 93 (82.3%) had a history of platinum‐based chemotherapy, and the others had a history of treatment with cytotoxic monotherapy. In the 18 patients positive for molecular biomarkers, 17 (94.4%) had a history of therapy involving a molecular‐targeting agent. The remaining one patient carried a de novo *EGFR* T790M mutation and did not receive *EGFR* tyrosine kinase inhibitor therapy due to a good response to platinum‐based chemotherapy, followed by nivolumab treatment.

**Table 1:** Patient characteristics

Characteristics		N	%
No. of patients		113	
Median age (range)		68.5 (36–86)^†^	
Sex	Male/Female	87/26	77.0/23.0
Histology	Adenocarcinoma	56	49.5
	Squamous cell carcinoma	47	41.6
	Adenosquamous carcinoma	1	0.9
	Other	9	8.0
Stage (TNM eighth edition)	III	38	33.6
	IVA	25	22.1
	IVB	31	27.4
	Postoperative recurrence	19	16.8
*EGFR*/*ALK* mutation		18	15.9
PD‐L1 TPS	Untested	53	46.9
	<1%	27	24.0
	1–49%	24	21.2
	≥50%	9	7.9
ECOG PS	0	19	16.8
	1	74	65.5
	2	19	16.8
	3	1	0.9
	4	0	0
No. of previous therapies	1	51	45.1
	2	25	22.1
	**≥**3	37	32.7
Laboratory tests and scores			
ANC	Cells/mm^3^	5717 ± 4759^‡^	
ALC	Cells/mm^3^	1126 ± 442^‡^	
Creatinine	mg/dL	0.95 ± 0.86^‡^	
CRP	mg/dL	3.30 ± 4.70^‡^	
Alb	g/dL	3.56 ± 0.55^‡^	
NLR		6.3 ± 7.2^‡^	
	≥5.8	39	34.5
	<5.8	74	65.5
CAR		1.13 ± 0.19^‡^	
	≥0.83	39	34.5
	<0.83	74	65.5
Modified GPS	0	39	34.5
	1	37	32.7
	2	37	32.7
Response and survival			
Best overall response	CR	0	0
	PR	27	23.9
	SD	26	23.0
	PD	50	44.2
	NE	10	8.9
Response rate	ORR (%), CI	26.2	0.18–0.36^§^
	DCR (%), CI	51.5	0.41–0.61^§^
Survival time	mPFS (month), CI	2.3	1.6–2.5^§^
	mOS (month), CI	14.0	9.9–17.4^§^
No. of mortality events	One‐year	48	42.5
	Total	66	58.4

^†^ Median (range).

^‡^ Mean ± standard deviation.

^§^ 95% CI.

ALC, absolute lymphocyte counts; ANC, absolute neutrophil counts; ALK, anaplastic lymphoma kinase; CAR, C‐reactive protein to albumin ratio; CI, confidence interval; CR, complete response; CRP, C‐reactive protein; DCR, disease control rate; ECOG PS, Eastern Cooperative Oncology Group performance status; EGFR, epidermal growth factor receptor; GPS, Glasgow prognostic score; mOS, median overall survival; mPFS, median progression‐free survival; NE, not evaluated; NLR, neutrophil to lymphocyte ratio; ORR, objective response rate; PD, progressive disease; PD‐L1 TPS, programmed death‐ligand 1 tumor proportion score; PR, partial response; SD, stable disease.

### Evaluation of NLR, CAR, and modified GPS

The optimal cutoff values for the NLR and CAR according to the AUCs were determined as 5.8 and 0.83, respectively. Baseline laboratory test results are shown in Table 1. Modified GPS scores of 0, 1, and 2 were present in 39 (34.5%), 37 (32.7%), 37 (32.7%) patients, respectively. The mean NLR was 6.30, with a NLR <5.8 observed in 74 patients (65.5%) and ≥5.8 in 39 patients (34.5%). The mean CAR was 1.13, with a CAR <0.83 observed in 74 patients (65.5%) and ≥0.83 in 39 patients (34.5%). CARs and NLRs collected eight weeks after initiation of nivolumab therapy were evaluated in 74 (65.5%) and 81 (71.7%) patients, with time‐series changes in CAR and NLR divided into groups as patients showing a partial response (PR) or progressive disease (PD), as shown in Fig 3. In the PR group, time‐dependent changes in CAR (Fig 3a) and NLR (Fig 3b) were assessed in 20 and 23 patients, respectively, revealing a nonsignificant change in NLR (*P* = 0.142), whereas CAR levels declined significantly over the eight‐week period (*P* = 0.0023). In the PD group, changes in both CAR (Fig 3c) and NLR (Fig 3d) assessed in 28 patients, indicated significant elevations in both over the eight‐week period (*P* = 0.038 and *P* = 0.0095, respectively).

### Treatment response

Table 1 shows patient response to treatment. No patients in the cohort experienced complete remission as the best overall response, and 10 (8.9%) patients were not evaluated as their treatment response could not be confirmed. The median number of cycles of nivolumab administration was five (range: 1–65), and the ORR and DCR were 26.2% (95% confidence interval (CI) 0.18–0.36) and 51.5% (95% CI: 0.41–0.61), respectively.

### Survival analysis

**Figure 1:**
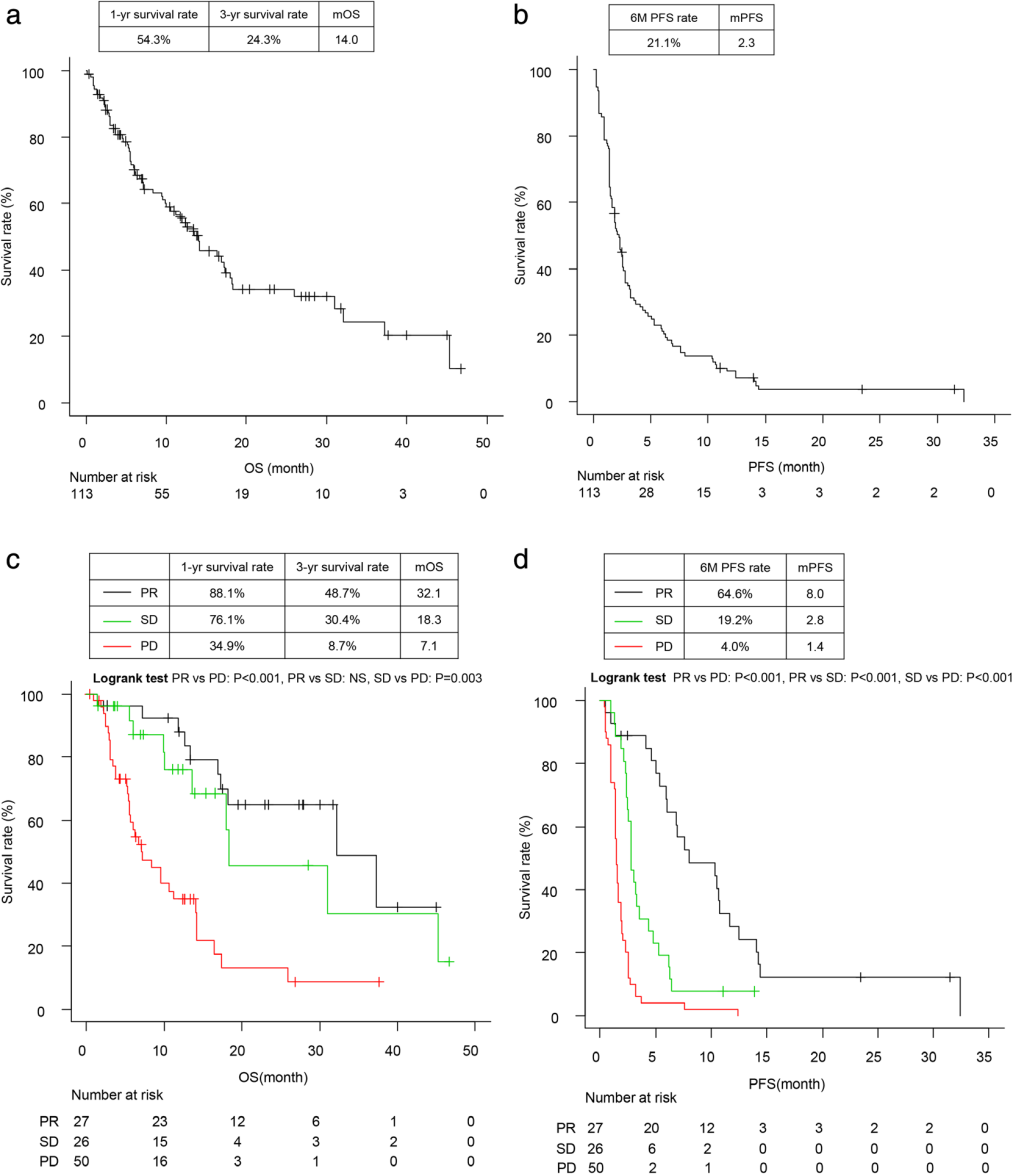
(**a**,**b**) Overall survival (OS) and progression‐free survival (PFS) analysis of the study cohort. Kaplan‐Meier survival curves for (**a**) OS and (**b**) PFS. Median OS and PFS were 14.0 months and 2.3 months, respectively. (**c**,**d**) OS and PFS according to response to nivolumab therapy. Kaplan–Meier survival curves for (**c**) OS and (**d**) PFS according to treatment response. The black line represents the partial response (PR) group, the green line represents the stable disease (SD) group, and the red line represents the progressive disease (PD) group. (**c**) (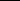) PR, (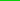) SD, (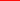) PD; (**d**) (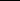) PR, (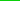) SD, (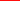) PD

**Figure 2:**
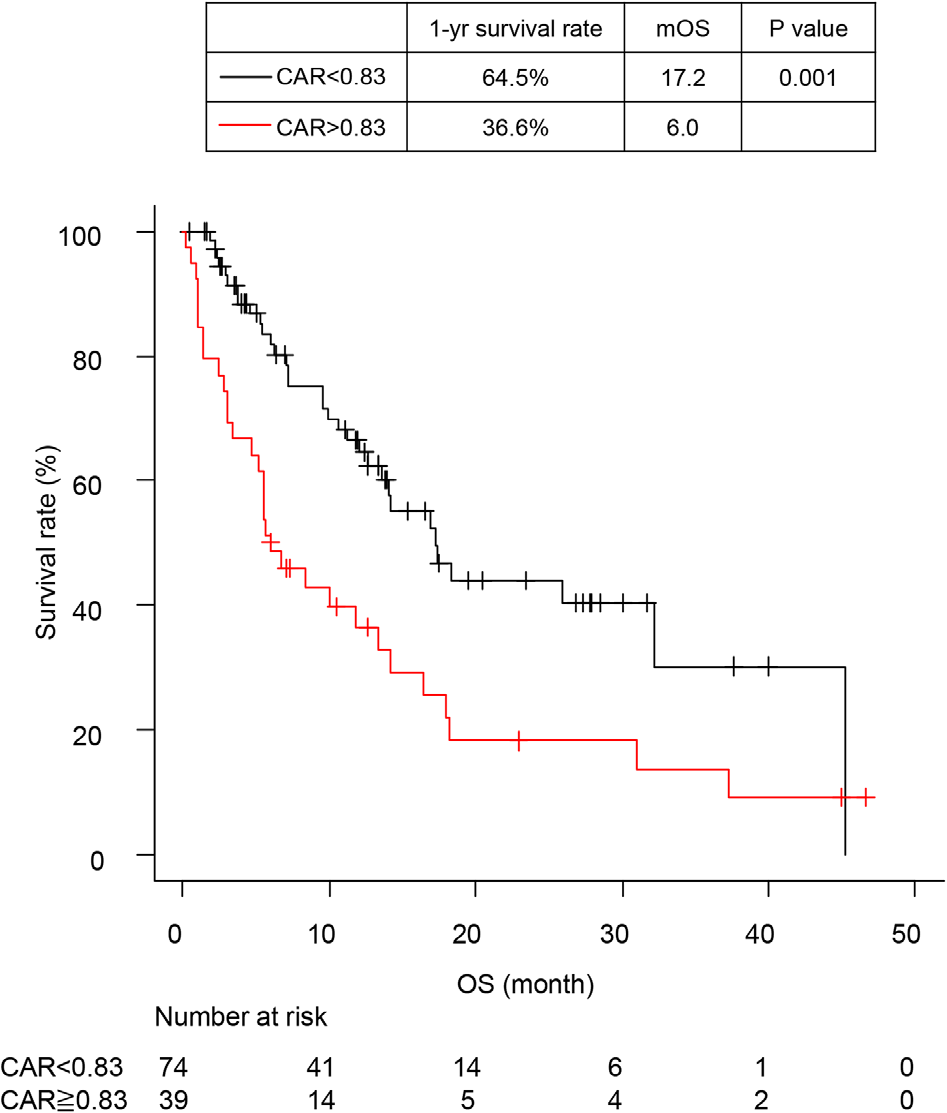
Overall survival (OS) analysis according to the baseline C‐reactive protein:albumin ratio (CAR). Kaplan–Meier survival curves for OS according to CARs. The black line represents a baseline CAR <0.83, and the red line represents a baseline CAR ≥0.83. (**c**) (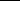) CAR <0.83, (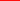) CAR ≥0.83

The median OS and PFS were 14.0 months (95% CI: 9.9–17.4) (Fig 1a) and 2.3 months (95% CI: 1.6–2.5) (Fig 1b), respectively. Additionally, there were 48 (42.5%) one‐year mortality events and 66 (58.4%) total mortality events during the study period, with three‐ and six‐month PFS rates of 34.9% and 21.1%, respectively. Comparison of survival times between each treatment‐response group indicated that patients in the PR group had significantly longer OS and PFS than those in the PD group (Fig 1c,d). The one‐year OS rates in the PR group and PD group were 88.1% and 34.9%, and the three‐year OS rates were 48.7% and 8.7%, respectively (Fig 1c), and the three‐month PFS rates in the two groups were 88.9% and 10.0%, whereas the six‐month PFS rates were 64.6% and 4.0%, respectively (Fig 1d). Logistic regression analysis using a univariate model for one‐year mortality events revealed age ≤70 (odds ratio [OR] 2.74, *P* = 0.017), an ECOG PS score of 2 or 3 (OR 3.38, *P* = 0.019), a baseline NLR ≥5.8 (OR 3.19, *P* = 0.005), and a baseline CAR ≥0.83 (OR 3.78, *P* = 0.001) as the statistically significant variables. Moreover, multivariate analysis demonstrated that a baseline CAR ≥0.83 (OR 3.42, 95% CI 1.09–10.7, *P* = 0.034) was the only significant factor for one‐year mortality events (Table 2). Univariate analysis demonstrated age ≤70 (hazard ratio (HR) = 2.32, *P* = 0.005), ECOG PS score of 2 or 3 (HR = 3.55, *P* < 0.001), modified GPS score of 2 (HR = 1.80, *P* = 0.028), baseline NLR ≥5.8 (HR = 2.47, *P* < 0.001), and baseline CAR ≤0.83 (HR = 2.23, *P* = 0.001) as parameters associated with inferior OS. Further, multivariate analysis using a Cox proportional hazards models revealed age ≤70 (HR = 2.04, 95% CI: 1.20–3.71, *P* = 0.019), ECOG PS score of 2 or 3 (HR = 2.68, 95% CI: 1.39–5.18, *P* = 0.003), and baseline CAR ≥0.83 (HR = 1.82, 95% CI: 1.04–3.19, *P* = 0.037) as associated with inferior OS (Table 3) (Fig 2).

**Table 2:** Logistic regression analysis for one‐year mortality events (*N* = 113, 48 of which were one‐year death events)

		Univariate model		Multivariate model	
Variables		OR (95% CI)	*P*‐value	OR (95% CI)	*P*‐value
Age	≤70 vs. >70	2.74 (1.19–6.29)	0.017	2.30 (0.94–5.66)	0.07
Sex	Female vs. male	1.34 (0.55–3.23)	0.52		
Histology	Sq vs. non‐Sq	0.72 (0.34–1.56)	0.41		
*EGFR*/*ALK* mutations	With vs. without	1.20 (0.43–3.32)	0.73		
ECOG PS	2–3 vs. 0–1	3.38 (1.23–9.29)	0.019	2.73 (0.88–8.43)	0.081
No. of previous therapies	≥2 vs. 1	1.12 (0.53–2.38)	0.77		
Modified GPS	2 vs. 0–1	1.97 (0.89–4.34)	0.093	0.71 (0.23–2.20)	0.56
Baseline NLR	≥5.8 vs. <5.8	3.19 (1.42–7.14)	0.005	1.94 (0.78–4.82)	0.16
Baseline CAR	≥0.83 vs. <0.83	3.78 (1.67–8.55)	0.001	3.42 (1.09–10.70)	0.034

ALK, anaplastic lymphoma kinase; CAR, C‐reactive protein to albumin ratio; CI, confidence interval; EGFR, epidermal growth factor receptor; ECOG PS, Eastern Cooperative Oncology Group performance status; GPS, Glasgow prognostic score; NLR, neutrophil to lymphocyte ratio; OR, odds ratio; Sq, squamous.

**Table 3:** Cox proportional hazards models for OS (*N* = 113, including 66 death events)

		Univariate analysis		Multivariate analysis	
Variables		HR (95% CI)	*P*‐value	HR (95% CI)	*P*‐value
Age	≤70 vs. >70	2.32 (1.29–4.17)	0.005	2.04 (1.20–3.71)	0.019
Sex	Female vs. male	1.16 (0.65–2.07)	0.62	1.34 (0.73–2.44)	0.35
Histology	Sq vs. non‐Sq	0.81 (0.49–1.34)	0.41		
*EGFR*/*ALK* mutations	With vs. without	1.21 (0.64–2.28)	0.56	1.42 (0.71–2.85)	0.32
ECOG PS	2–3 vs. 0–1	3.55 (1.94–6.50)	<0.001	2.68 (1.39–5.18)	0.003
No. of previous therapies	≥2 vs. 1	1.13 (0.68–1.87)	0.65		
Modified GPS	2 vs. 0–1	1.80 (1.07–3.04)	0.028		
Baseline NLR	≥5.8 vs. <5.8	2.47 (1.51–4.05)	<0.001	1.64 (0.94–2.86)	0.085
Baseline CAR	≥0.83 vs. <0.83	2.23 (1.36–3.65)	0.001	1.82 (1.04–3.19)	0.037

ALK, anaplastic lymphoma kinase; CAR, C‐reactive protein to albumin ratio; CI, confidence interval; ECOG PS, Eastern Cooperative Oncology Group performance status; EGFR, epidermal growth factor receptor; GPS, Glasgow prognostic score; HR, hazard ratio; NLR, neutrophil to lymphocyte ratio; Sq, squamous.

## Discussion

**Figure 3:**
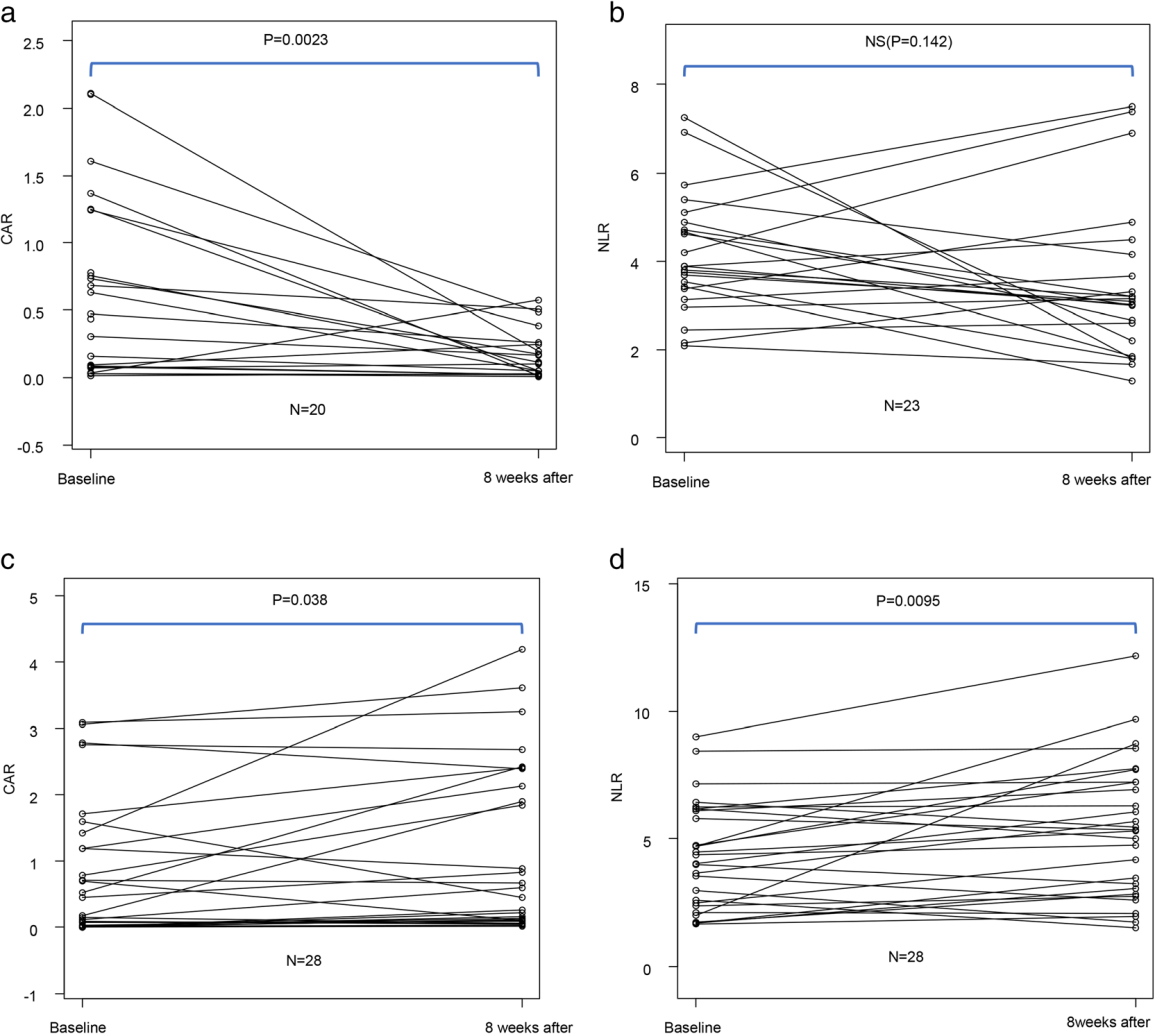
Time‐dependent changes in the C‐reactive protein:albumin ratio (CAR) and neutrophil:lymphocyte ratio (NLR) in the (**a**,**b**) partial response (PR) and (**c**,**d**) progressive disease (PD) groups. Time‐dependent changes (from baseline values to those eight weeks after starting nivolumab treatment) in the (**a**,**c**) CAR and (**b**,**d**) NLR according to the Wilcoxon signed‐rank test.

In this retrospective analysis, we evaluated the clinical characteristics and prognosis of 113 eligible patients with NSCLC, treated with nivolumab monotherapy, at two institutes. Our data show clinical response rates, one‐year survival rates, and OS and PFS rates comparable to those in previous clinical trials.^17^ In patients achieving PR, we found that the baseline NLR and CAR decreased at eight weeks after the introduction of nivolumab, with the decrease in CAR as statistically significant (Fig. 3a). Moreover, of the clinical markers investigated, an elevated baseline CAR was a significant factor associated with one‐year mortality and OS according to multivariate analysis (Tables 2 and 3).

Nivolumab is an ICI which is approved for treatment of NSCLC patients and widely used in clinical practice; however, specific prognostic biomarkers for nivolumab therapy have not yet been identified. Histopathological markers, such as (PD‐L1) levels in tumor tissue,^18^ tumor mutation burden,^19^ and increased CD8+ tumor‐infiltrating lymphocytes,^20^ are reportedly predictive of the efficacy of anti‐PD‐1/PD‐L1 therapy in NSCLC patients. However, these markers are inconvenient because they require access to tumor tissue for testing and cannot be evaluated as a time series; therefore, it is crucial to identify clinical markers capable of noninvasive evaluation via routine laboratory methods. Importantly, our findings identify CAR as a potential predictive and/or prognostic factor for nivolumab efficacy in NSCLC patients. To the best of our knowledge, this is the first study demonstrating association of CAR with one‐year mortality and OS in NSCLC patients treated with nivolumab monotherapy, as well as its potential prognostic efficacy.

Serum CRP and albumin levels are prognostic markers in multiple cancer types. CRP is a positive acute‐phase protein; its synthesis is induced in the liver by IL‐6, and is associated with an inflammatory and infectious process.^21^ By contrast, albumin is both a nutritional marker and a negative acute‐phase protein with attenuated production under inflammatory conditions.^22^ Therefore, modified GPS and CAR might represent markers of systemic inflammation, as well as the dynamics associated with the tumorigenic inflammatory response. This suggests that a higher modified GPS and CAR reflect upregulation of proinflammatory cytokine levels (IL‐6 and IL‐1) reportedly involved in tumorigenic inflammation and associated with resistance to anticancer agents.^23^, ^24^

CAR is used to evaluate patient outcomes related to acute medical conditions and sepsis.^25^, ^26^ Recently, the prognostic efficacy of CAR has been reported in patients with several types of cancer.^27^, ^28^, ^29^, ^30^, ^31^ For lung cancer, the prognostic value of CAR in NSCLC has been reported in patients receiving either platinum‐based or nonplatinum‐based chemotherapy,^32^ as well as in patients with advanced small cell lung cancer.^33^ Additionally, a retrospective study showed that elevated CAR levels were significantly associated with early death events in NSCLC patients treated with nivolumab (death within three months).^34^ Although modified GPS has prognostic utility in certain cancers, it was not a significant factor in the present study, whereas the baseline CAR value is a significant factor for one‐year mortality events and poor OS in our study. A possible explanation might be that CAR is a continuous variable as compared to modified GPS, which is an ordinal variable scored in three groups.

Additionally, we identified significant time‐dependent alterations in CAR between patients experiencing PR and PD. Data collected eight weeks after initiation of nivolumab therapy demonstrated a significant decrease in CAR in the PR group and an increase in the PD group. Although several studies have reported that time‐series changes in hematological cell‐based markers are predictive of outcomes in NSCLC patients treated with nivolumab,^35^, ^36^ the present study is the first to show serial changes in CAR. Changes in these inflammatory markers following initiation of treatment indirectly reflect improved inflammatory dynamics in the tumor microenvironment. A previous study reported that survival‐time analysis demonstrated significantly better PFS and OS in the PR group relative to the PD group.^3^ In the present study, the PR group achieved a durable response to nivolumab treatment resulting in a longer survival time. Moreover, our study found that a decrease in CAR following nivolumab therapy may be predictive of long‐term therapeutic efficacy.

The present study has several limitations. It is a retrospective study which was conducted in two facilities with a relatively small sample size, which may have influenced the results associated with NLR, previously reported as a predictive marker of nivolumab efficacy but did not show significant results in our cohort. Additionally, time‐dependent changes in CAR could only be assessed in a limited number of patients; therefore, these findings cannot be generalized to all NSCLC patients treated with nivolumab. Precise information regarding patient status before and after nivolumab therapy was not included in this study. Previous treatments may have affected laboratory test results and background factors for patients at the beginning of nivolumab therapy, whereas the post‐treatment status might also have affected the subsequent survival time. Finally, the utility of PD‐L1, which is a prognostic factor associated with nivolumab treatment, was not evaluated in this study because 46.9% of the patients had not been tested for PD‐L1 in our cohort.

In conclusion, we found that the baseline CAR could be a useful clinical biomarker of one‐year survival and OS time in patients with NSCLC receiving nivolumab therapy. Furthermore, the decline in CAR at eight weeks after the first administration of nivolumab might indicate a durable response. Importantly, our findings showed that NLR and modified GPS were not predictive of treatment response or prognosis. CAR can be derived from simple and repeatable laboratory tests; therefore, this measurement could be potentially useful for patients treated with nivolumab, although further investigations are needed to verify the predictive and prognostic values of CAR.

## Disclosure

Author Dr Shintaro Kanda has the following financial relationships to disclose (relevant financial activities outside the submitted work): AstraZeneca, grant and personal fees; Abbvie, grant and personal fees; Ono Pharmaceutical, grant and personal fees; Bristol‐Myers Squibb, grant and personal fees; MSD, grant and personal fees; Chugai Pharmaceutical, grant and personal fees; Cimic Shift Zero, grant and personal fees.

The rest of the authors have no declarations of interest.
